# Effect of Annealing on Microstructure and Mechanical Properties of Al_0.5_CoCrFeMo_x_Ni High-Entropy Alloys

**DOI:** 10.3390/e20110812

**Published:** 2018-10-23

**Authors:** Yan-Xin Zhuang, Xiu-Lan Zhang, Xian-Yu Gu

**Affiliations:** Key Laboratory of Electromagnetic Processing of Materials, Ministry of Education, Northeastern University, Shenyang 110819, China

**Keywords:** high-entropy alloys, annealing, microstructure, mechanical properties, phase constituent

## Abstract

The effect of annealing temperature on the microstructure, phase constituents and mechanical properties of Al_0.5_CoCrFeMo_x_Ni high-entropy complex alloys has been investigated at a fixed annealing time (10 h). The 600 °C-annealing has no obvious effect on their microstructures, while the annealing at 800–1200 °C enhances the precipitation of (Al,Ni)-rich ordered BCC phase or/and (Cr,Mo)-rich σ phase, and thereby greatly affects the microstructure and mechanical properties of the alloys. All the annealed Al_0.5_CoCrFeNi alloys are composed of FCC and (Al,Ni)-rich ordered BCC phases; the phase constituent of the Al_0.5_CoCrFeMo_0.1_Ni alloy changes from FCC + BCC (600 °C) to FCC + BCC + σ (800 °C) and then to FCC + BCC (1100 °C); the phase constituents of the Al_0.5_CoCrFeMo_0.2_Ni and Al_0.5_CoCrFeMo_0.3_Ni alloys change from FCC + BCC + σ to FCC + BCC with the annealing temperature rising from 600 to 1200 °C; while all the annealed Al_0.5_CoCrFeMo_0.4_Ni and Al_0.5_CoCrFeMo_0.5_Ni alloys consist of FCC, BCC and σ phases. The phase constituents of most of the alloys investigated are in good agreement with the calculated results from Thermo-Calc program. The alloys annealed at 800 °C under current investigation conditionshave relative fine precipitations and microstructure, and thereby higher hardness and yield stress.

## 1. Introduction

The recently developed high-entropy alloys (HEAs), also known as multi-principal elements alloys or complex concentrated solid solution alloys, have attracted increasing attention due to their unique microstructures and adjustable properties [1,2,3,4,5,6]. The HEAs, which contains more than five principal elements with concentrations from 5 to 35 at.% for each principal element [2], tend to form simple solution structures (FCC, BCC, HCP or mixed) rather than many complex phases. To date, many efforts have been made to understand and control the structure and properties within as-cast and/or homogenized HEAs [7,8,9,10,11,12,13,14,15]. The original concept of HEAs and the strict restriction on the HEA design strategy proposed by Yeh [2] have also been relaxed. Depending on their composition, the as-cast and/or homogenized HEAs can have many interesting mechanical and physical properties, and wide potential applications.

It has been realized that the solid solutions in as-cast HEAs are the firstly formed solid phases upon cooling from the molten liquids, and are generally metastable phases at room temperature [16]. The room-temperature-metastable solid solutions could transform to other phases at an appropriate annealing temperature, and the annealing process can modify the microstructure and properties of the HEAs. Aging the AlCoCrCuFeNi HEA at elevated temperatures causes the structure gradually to transform from stabilized BCC to FCC, decreases the yield strength of the alloy, and increases the plastic strain of the material [17]. Aging the Cu_0.5_CoCrFeNi alloy at 1100–1350 °C induces the precipitation of a Cr-rich phase in the FCC matrix of the alloy, and improves the anti-corrosion properties of the alloy [18]. The AlNi-based B2 phase and Cr-rich σ phase formed in the Al_0.5_CrFeCoNiCu HEAs annealed at 700–900 °C [19]. A needle-like Cu-rich FCC phase has been precipitated from the BCC dendrite region at the annealing temperatures higher than 973 K in the AlCoCuFeNi HEA [20]. The microstructure and properties of the Al_x_CoCrFeNi HEAs have a strong dependence on the Al content and annealing temperature [21]. The phase stability of the metastable solid solutions is actually becoming a critical issue for HEAs.

Over the last decades, the single-FCC or BCC HEAs have been intensively investigated and developed. In fact, it is very hard to reach a balance between high strength and high ductility for the single-phase HEAs due to the fact that the single-FCC HEAs normally have good ductility and poor strength, while the single-BCC HEAs generally have poor ductility and good strength. By adjusting the composition of the alloys, a eutectic high-entropy alloy AlCoCrFeNi_2.1_ with a regular FCC/BCC lamellar structure has been developed, and the alloy has an excellent combination of high strength and high ductility [22], which was attributed to the coupling between the ductile FCC and brittle BCC phases during tension deformation [23]. A transformation-induced plasticity-assisted dual-phase (TRIP-DP) HEA has also been developed, in which two high-entropy phases (FCC γ matrix and laminate HCP ε phase) present [24]. The TRIP-DP HEA combines the solid-solution strengthening effect in HEAs with the TRIP effect, exhibits multiple deformation mechanisms and dynamic strain partitioning behavior [25], and has improved strength and ductility compared to its corresponding single-phase HEA. This combined increases in strength and ductility distinguishes the TRIP-DP-HEA alloy from other structural materials [24]. All these results have showed that the dual- or multi-phase HEAs can display an excellent combination between strength and ductility, and are becoming the future direction for designing advanced HEAs.

The Al_0.5_CoCrFeNi alloy has a mixed FCC + B2 dual-phase structure with FCC as the dominant structure [7,21]. Our previous results have shown that the addition of Mo into this alloy system could enhance the formation of the σ phase, and tune the mechanical properties of the as-cast Al_0.5_CoCrFeMo_x_Ni [26]. Although we have evaluated the high-temperature equilibrium phases existing in Al_0.5_CoCrFeMo_x_Ni alloy using thermodynamic calculation, the results need to be assured by experiments. On the other hand, an appropriate annealing process can further modify the microstructure and improve its mechanical properties. In order to understand the temperature effect on this HEA system, the aged-and-quenched microstructure, phase transformation and mechanical properties have been investigated. The pseudo binary phase diagram derived from Thermo-Calc program has been compared with the experimental results.

## 2. Materials and Methods

Ingots with nominal compositions of Al_0.5_CoCrFeMo_x_Ni (x = 0, 0.1, 0.2, 0.3, 0.4 and 0.5, denoted by Mo_0_, Mo_0.1_, Mo_0.2_, Mo_0.3_, Mo_0.4_ and Mo_0.5_, respectively) were prepared by arc-melting the mixture of constituent elements with purity better than 99.9 wt.% in a water-cooled copper hearth under a titanium-gettered high-purity argon atmosphere. The ingots were remelted at least four times to assure their chemical homogeneity. Samples for microstructural observation and annealing processes were cut from the ingots, mechanically ground and polished through standard routines. Some samples were sealed in quartz tubes under a vacuum better than 1 × 10^−2^ Pa, and then annealed at given temperatures for 10 h. After the annealing process, the samples were quenched in water. The phase constitutions of the alloys were characterized using X-ray diffraction (XRD) with Cu K_α_ radiation (X’Pert Pro, PANalytical B.V., Almelo, The Netherlands). The microstructure and chemical compositions were examined on the polished samples using scanning electron microscopy (SEM, SSX-550, SHIMADZU corp., Kyoto, Japan) equipped with energy dispersive spectrometry (EDS). Hardness was characterized using a Vickers hardness tester (Wolpert 452SVD, Wolpert Wilson instrument, Shanghai, China) under a load of 5 Kgf for 15 s. The hardness measurements were made on at least seven points to yield an average value for each sample. Room temperature compressive tests were performed on cylindrical specimens with 5 mm in diameter and 10 mm in length using a SHIMADZU precision universal tester (AG-X, SHIMADZU corp., Kyoto, Japan) with a strain rate of 8.3 × 10^−4^ s^−1^. 

Thermodynamic calculations were conducted using the Thermo-Calc TCCS program in conjunction with a commercial TTNI7 database, which is developed for Ni-based alloys and contains 22 elements including Fe, Co, Ni, Al, Cr, Cu, Nb and Mo. The pseudo binary phase diagram, fraction and composition of each equilibrium phase at different temperatures were derived using the program.

## 3. Results and Discussion

### 3.1. Phase Transformation of Annealed Al_0.5_CoCrFeMo_x_Ni Alloys

**Figure 1:**
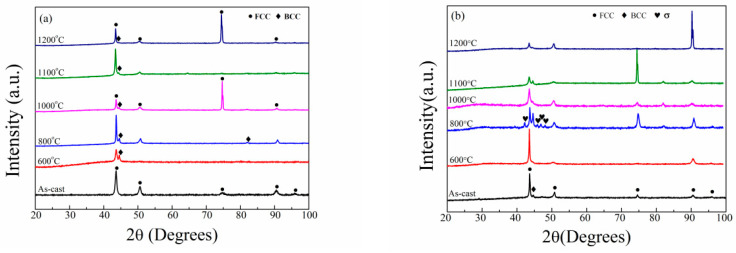

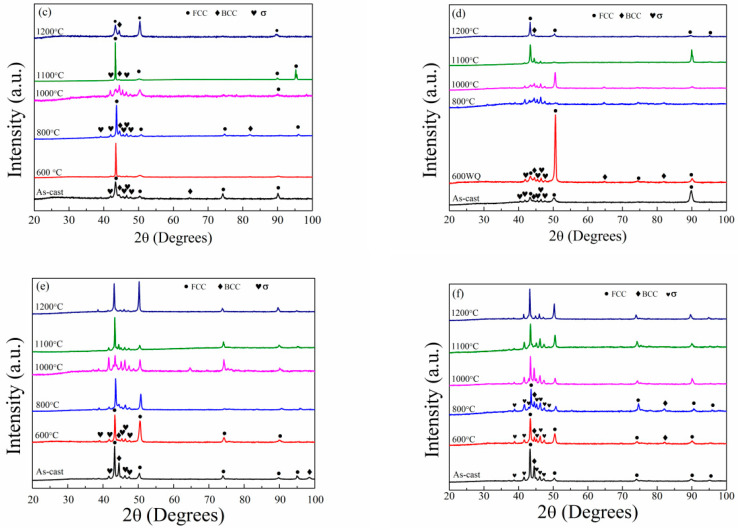
X-ray diffraction (XRD) patterns of the as-cast Al_0.5_CoCrFeMo_x_Ni alloys and the alloys annealed at different temperatures for 10 h followed by a water quenching. (**a**) x = 0; (**b**) x = 0.1; (**c**) x = 0.2; (**d**) x = 0.3; (**e**) x = 0.4; (**f**) x = 0.5.

Figure 1 summaries the XRD patterns of the Al_0.5_CoCrFeMo_x_Ni multicomponent high-entropy alloys annealed at different temperatures for 10 h followed by water quenching. The XRD patterns of the as-cast alloys are also given in the figure. The addition of Mo and the annealing process affect the phase constituents in the alloys. The addition of Mo enhances the formation of σ and ordered BCC phases in the as-cast Al_0.5_CoCrFeMo_x_Ni alloys as reported in our previous work [26]. The Bragg peaks in the XRD patterns of the as-cast Al_0.5_CoCrFeNi alloy can be indexed to a simple FCC phase. When the Al_0.5_CoCrFeNi alloy was annealed at the temperatures between 600–1200 °C for 10 h, the Bragg peaks corresponding to a BCC phase can found on the XRD patterns even though the peak at 2θ of about 44 is very weak for the alloy annealed at 1200 °C, meaning that the annealing induces the formation of the BCC phase. Similarly, the as-cast and 600 °C-annealed Mo_0.1_ alloys have a dominant FCC phase and a minor BCC phase, while a σ phase has been found in the Mo_0.1_ alloys annealed at 800 °C and disappears again in the alloys annealed at 1000–1200 °C. The as-cast Mo_0.2_ alloy and the alloys annealed at 600–1100 °C consist of three phases, namely FCC, BCC and σ phases, while the Mo_0.2_ alloys annealed at 1200 °C only have FCC and BCC phases. The as-cast Mo_0.3_ alloy and the alloys annealed at 600–1100 °C are composed of the FCC, BCC and σ phases, while only FCC and BCC phases can be found in the alloy annealed at 1200 °C. The as-cast and annealed Mo_0.4_ alloys have the FCC, BCC and σ phases even though the peaks for BCC phase are very weak in the alloy annealed at 1200 °C. The as-cast Mo_0.5_ alloy and the alloys annealed at 600–1200 °C have FCC, BCC and σ phases even though the peaks for the BCC phase are very weak in the alloy annealed at 1200 °C. It can be concluded that both the Mo content and the annealing temperature affect the phase constituents of the Al_0.5_CoCrFeMo_x_Ni alloys. On the other hand, some kind of preferred orientation can also be observed in some of the alloys.

### 3.2. Microstructures of Annealed Al_0.5_CoCrFeMo_x_Ni Alloys

**Figure 2:**
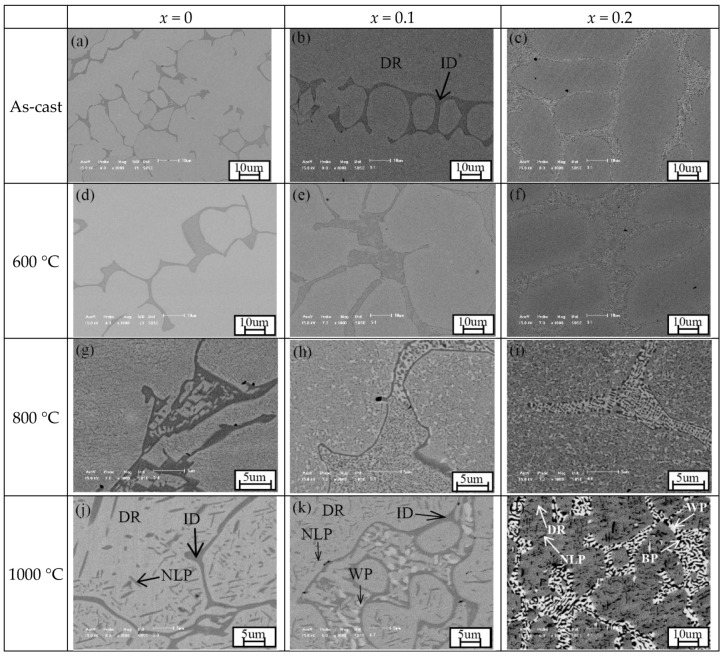

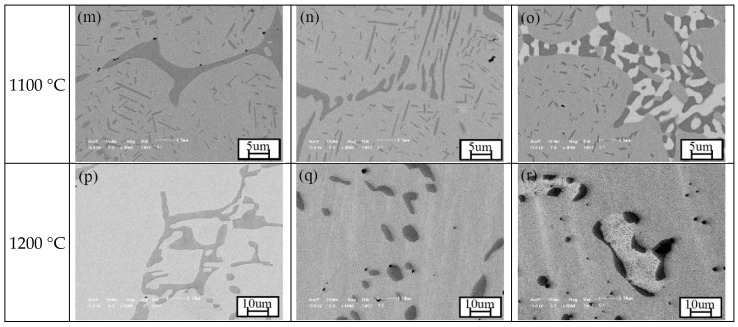
Back-scattering scanning electron microscope (SEM) images of the as-cast Mo_0_, Mo_0.1_ and Mo_0.2_ high entropy alloys and the alloys annealed at different temperatures for 10 h. DR is for FCC dendrite phase, ID is for (Al,Ni)-rich interdendrite phase, NLP is for needle-like (Al,Ni)-rich recipitate, WP is for white (Cr,Mo)-rich precipitate, and BP is for black (Al,Ni)-rich precipitate. (**a**) as-cast Mo_0_ alloy; (**b**) as-cast Mo_0.1_ alloy; (**c**) as-cast Mo_0.2_ alloy; (**d**) Mo_0_ alloy annealed at 600 °C; (**e**) Mo_0.1_ alloy annealed at 600 °C; (**f**) Mo_0.2_ alloy annealed at 600 °C; (**g**) Mo_0_ alloy annealed at 800 °C; (**h**) Mo_0.1_ alloy annealed at 800 °C; (**i**) Mo_0.2_ alloy annealed at 800 °C; (**j**) Mo_0_ alloy annealed at 1000 °C; (**k**) Mo_0.1_ alloy annealed at 1000 °C; (**l**) Mo_0.2_ alloy annealed at 1000 °C; (**m**) Mo_0_ alloy annealed at 1100 °C; (**n**) Mo_0.1_ alloy annealed at 1100 °C; (**o**) Mo_0.2_ alloy annealed at 1100 °C; (**p**) Mo_0_ alloy annealed at 1200 °C; (**q**) Mo_0.1_ alloy annealed at 1200 °C; (r) Mo_0.2_ alloy annealed at 1200 °C.

Figure 2 presents the microstructure of the as-cast Mo_0_, Mo_0.1_ and Mo_0.2_ high entropy alloys and the alloys annealed at different temperatures for 10 h. Table 1 lists distribution of element in different phases of the alloys annealed at 1000 °C. The as-cast Mo_0_, Mo_0.1_ and Mo_0.2_ alloys have typical dendrite microstructures. The dendrite (DR) phase is FCC phase, while the interdendrite (ID) region consists of (Al,Ni)-rich phase, FCC phase and/or (Cr,Mo)-rich phase [26]. The 600 °C annealing has no obvious effect on the microstructure of the three alloys. However, the annealing at temperatures between 800 and 1200 °C has a distinct influence on the microstructure of the three alloys. The needle-like precipitations have been found in the Mo_0_ alloys annealed at 800–1100 °C, and the EDS shows that both the needle-like precipitation (marked as NLP in the figure) and the dark matrix phase in the ID region have high Al and Ni, can be regarded as a (Al,Ni)-rich phase (refer to the data in Table 1). The (Al,Ni)-rich phase have an ordered BCC (B2) structure [21,26]. When increasing annealing temperature, the number of needle-like precipitation decreases, while its size increases. When the annealing temperature is up to 1200 °C, the needle-like (Al,Ni)-rich precipitation disappears. It is clear that the two phases (FCC and (Al,Ni)-rich ordered BCC) exist in the Mo_0_ alloy annealed at temperatures of 600–1200 °C, which is in good agreement with the observation from XRD patterns. Similarly, many obvious precipitations have been found in the Mo_0.1_ and Mo_0.2_ alloys annealed at 800–1100 °C. By contrast with the Mo_0_ alloys, a new phase enriched with Cr and Mo (bright phase marked as WP) has been observed in the Mo_0.1_ and Mo_0.2_ alloys. The (Cr,Mo)-rich phase is the σ phase [26]. There are three phases, namely FCC matrix, needle-like (Al,Ni)-rich ordered BCC phase and round-like (Cr,Mo)-rich σ phases, existing in the Mo_0.1_ alloys annealed at 800–1000 °C and the Mo_0.2_ alloys annealed at 600–1100 °C, while there are two phases (FCC and (Al,Ni)-rich ordered BCC phases) in the Mo_0.1_ alloys annealed at 1100 and 1200 °C and the Mo_0.2_ alloy annealed at 1200 °C. It can also be observed that the (Cr,Mo)-rich σ phase exists in the DR and ID regions in the Mo_0.1_ and Mo_0.2_ alloys annealed at 800 °C, but only appears together with the (Al,Ni)-rich ordered BCC phase in the alloys annealed at higher temperatures. 

The alloys annealed at 800 °C have fine microstructures, and are supposed to have better mechanical properties. The fine microstructure can be attributed to solid-state decomposition of the B2 phase and FCC matrix. The phenomena can be often found in Al_x_CoCrFeNi alloys [27,28,29,30]. Heat treatment at 620 °C for the Al_0.3_CoCrFeNi alloy caused the transformation of FCC to FCC + L1_2_ or FCC + B2 + σ phases depending on the processing routes, and the microstructural variation realized by different process pathways was attributed to the competition between the thermodynamic driving force and activation barrier for the second-phase nucleation [27]. An aging process lead to the spinodal decomposition reactions of the FCC matrix and the interdendrite (Al,Ni)-rich phase in Al_0.5_CoCrFeNi alloy, and the phase segregation effect was explained based on the mixing enthalpy between different atom pairs [28]. Recently, Rao et al. found that a (Al,Ni)-rich L1_2_ phase was precipitated from the FCC matrix of Al_0.5_CoCrFeNi alloy, a Cr-rich BCC nanoprecipitate was observed in its B2 phase, and the Cr-rich BCC nanoprecipitate was the origin of the σ phase [29]. Banerjee et al. also reported that the solid-state decomposition resulted in the formation of FCC + L1_2_ and BCC + B2, accompanied by a compositional partitioning [30]. Cleary, the solid-state decomposition of the FCC matrix and (Al,Ni)-rich ordered BCC in the Mo-free Al_0.5_CoCrFeNi alloy is responsible for its fine structure at 800 °C. In the Mo-containing alloys, the addition of Mo enhances the formation of σ phase. Both Cr and Mo are strong σ-forming elements [31]. The σ phase forms either in a three-step formation from B2 region or directly from the FCC matrix [29].

**Figure 3:**
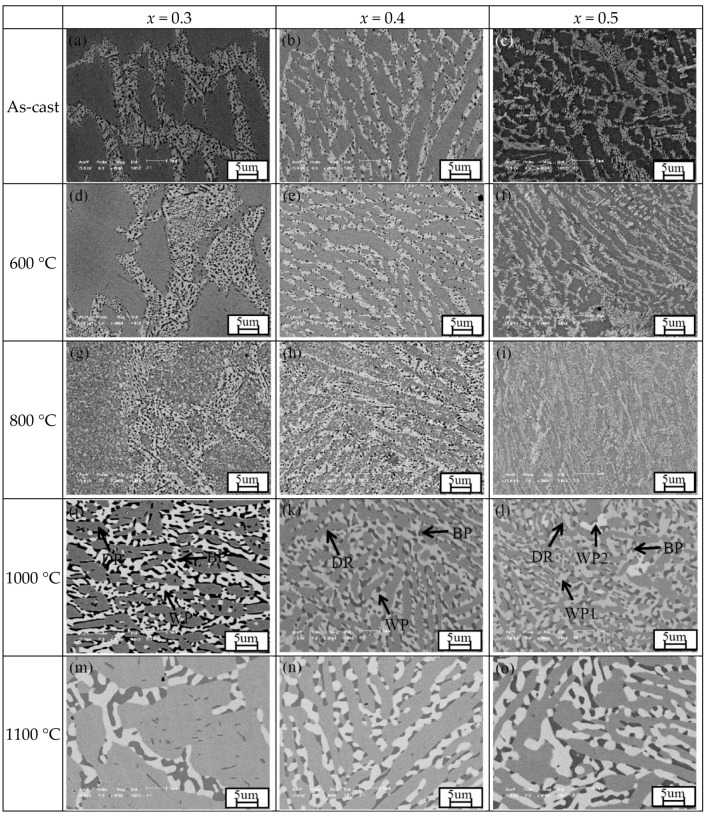

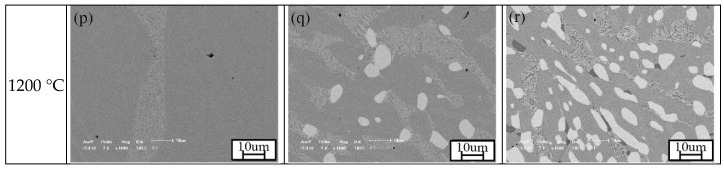
Back-scattering SEM images of the as-cast Mo_0.3_, Mo_0.4_, and Mo_0.5_ high entropy alloys and the alloys annealed at different temperatures for 10 h. DR is for dendrite FCC, WP1 is for white (Cr,Mo)-rich precipitate with relative low Mo content, WP2 is the whit (Cr,Mo)-rich precipitate with relative high Mo content, and BP is for black (Al,Ni)-rich precipitate. (**a**) as-cast Mo_0.3_ alloy; (**b**) as-cast Mo_0.4_ alloy; (**c**) as-cast Mo_0.5_ alloy; (**d**) Mo_0.3_ alloy annealed at 600 °C; (**e**) Mo_0.4_ alloy annealed at 600 °C; (**f**) Mo_0.5_ alloy annealed at 600 °C; (**g**) Mo_0.3_ alloy annealed at 800 °C; (**h**) Mo_0.4_ alloy annealed at 800 °C; (**i**) Mo_0.5_ alloy annealed at 800 °C; (**j**) Mo_0.3_ alloy annealed at 1000 °C; (**k**) Mo_0.4_ alloy annealed at 1000 °C; (**l**) Mo_0.5_ alloy annealed at 1000 °C; (**m**) Mo_0.3_ alloy annealed at 1100 °C; (**n**) Mo_0.4_ alloy annealed at 1100 °C; (**o**) Mo_0.5_ alloy annealed at 1100 °C; (**p**) Mo_0.3_ alloy annealed at 1200 °C; (**q**) Mo_0.4_ alloy annealed at 1200 °C; (r) Mo_0.5_ alloy annealed at 1200 °C.

Figure 3 summarizes the microstructure of the Mo_0.3_, Mo_0.4_ and Mo_0.5_ alloys at various annealing temperature. Table 2 lists the elemental distribution in each phase for the three alloys annealed at 1000 °C. All the three as-cast alloys are composed of FCC, (Al,Ni)-rich ordered BCC, and (Cr,Mo)-rich σ phases as stated in our previous work [26]. The 600 °C-annealing has also no obvious effect on the microstructure of the three alloys, while 800 °C-annealing induces the precipitation of a large amount of fine (Al,Ni)-rich BCC and (Cr,Mo)-rich σ phases in the alloys even though their typical dendrite microstructure remains. Obvious coarsening occurred when the alloys were annealed at 1000–1100 °C, and the morphology of BCC and σ phases changes greatly. The BCC and σ phases become larger, and most of them appear together, implying that certain crystallographic orientation exists between the ordered BCC and σ phases. A few precipitations with even higher Mo content can also be found in the Mo_0.5_ alloy annealed at 1000 °C (marked as WP2 in the corresponding image). When the alloys annealed at 1200 °C, the amount of ordered BCC and σ phases obviously decrease.

The wide composition range of each phase has been observed in the annealed alloys, as shown in Table 1 and Table 2. The addition of Mo into the Al_0.5_CoCrFeNi alloys can change the liquidus temperature or solvus temperature, the equilibrium phase composition, and even the formation sequence of the equilibrium phases [26]. The variable mixing enthalpy between different atoms and competition among the elements could be possible reasons for the variable composition of each phase. On the other hand, the needle-like (Al,Ni)-rich precipitate has a relatively small size, which might cause the inaccurately measured composition. Further work is going to clarify the reason for the wide composition. However, the wide composition range of each phase has no effect on the phase structure.

### 3.3. Calculated Pseudo Binary Phase Diagram and Its Comparison with Experiments

**Figure 4:**
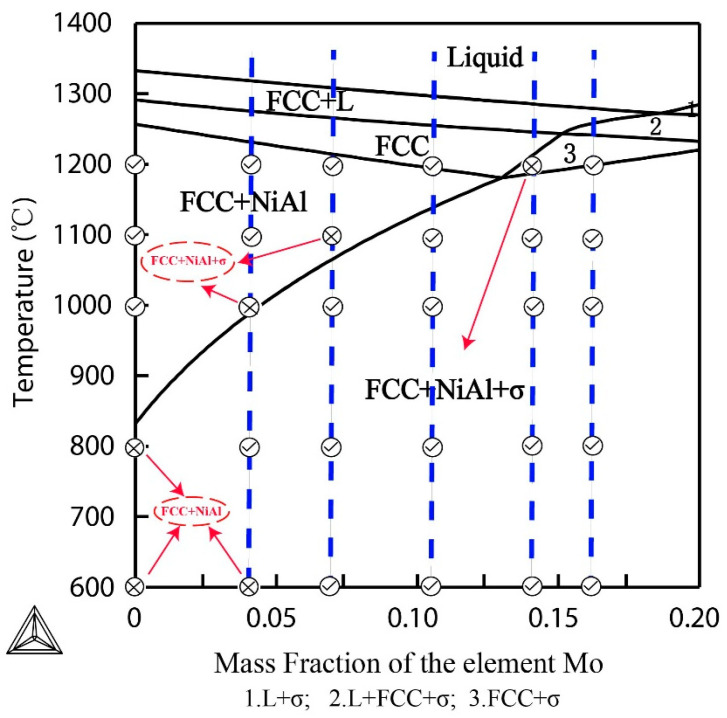
Pseudo binary phase diagram of Al_0.5_CoCrFeMo_x_Ni HEAs derived from Thermo-Calc. The dashed line represents the compositions of the alloys experimentally investigated in this work. The symbol ⊗ represents that the phase constituents of the alloys are different from the calculated results, and the text in red is the phase constituents of the alloys identified from experiments. The alloys marked with symbol 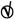 have the same phase constituents as calculated results.

It is clear that the phase constituents in the Al_0.5_CoCrFeMoxNi alloys depend on the content of Mo and the annealing temperature. Figure 4 is the calculated pseudo-binary phase diagram of Al_0.5_CoCrFeMo_x_Ni using the Thermo-Calc program, where the calculated equilibrium phase constituents in each region have been labeled. The alloys investigated in this work have also been marked in the diagram as the dash lines. The phase constituents in most of the alloys, which have been derived based on the XRD and microstructures above, are in good agreement with the calculated results. Only 6 samples among the 30 samples have a little difference with the calculated phase constituents from Thermo-Calc program. Careful examination found that the difference happened either near the boundary between different regions or at the low temperatures. For example, the Mo_0_ alloy at 800 °C, the Mo_0.1_ alloy at 1000 °C, the Mo_0.2_ alloy at 1100 °C and the Mo_0.4_ alloy at 1200 °C locate near the boundary between two regions. The difference might be attributed to the unspecified database for the alloy system, which makes the boundary line shift a little. Another reason could be the sluggish diffusion of elements in the high-entropy alloys, whereby the equilibrium state might not be reached in the annealing time used. The examples are the Mo_0_ and Mo_0.1_ alloys at 600 °C, where the calculated equilibrium phases in these two alloys are FCC, NiAl and σ phases, but only FCC and (Al,Ni)-rich ordered BCC phases have been identified from our experiments. Gwalani et al. reported that a different processing pathway resulted in different phases and the equilibrium phases in Al_0.3_CoCrFeNi alloy were FCC + B2 + σ phases [27]. Rao et al. also reported that the stable phases in Al_0.5_CoCrFeNi alloy are BCC + L1_2_ + σ phases [29]. Anyway, the calculated phase diagram could give us potential help in designing alloys and their corresponding annealing processes. 

### 3.4. Mechanical Properties of Annealed Al_0.5_CoCrFeMo_x_Ni Alloys

**Figure 5:**
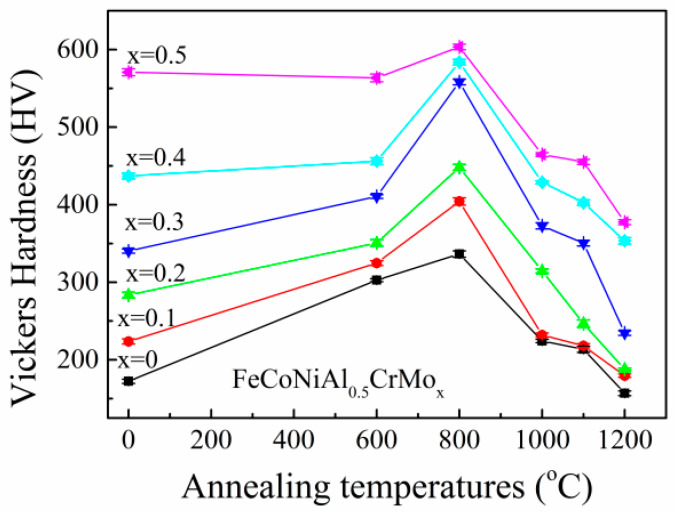
Hardness of Al_0.5_CoCrFeMo_x_Ni high-entropy alloys annealed at different temperatures for 10 h followed by water quenching.

Figure 5 depicts the room temperature hardness of the Al_0.5_CoCrFeMo_x_Ni high entropy alloys annealed at various annealing temperatures. The data at 25 °C represent the values of as-cast alloys. The hardness of the alloys increases with the increasing Mo, and this can be attributed to the lattice distortion and formation of BCC and σ phases. On the other hand, with annealing temperature rising, the hardness of the alloys first increases, reaches its maximum at 800 °C, and then decreases afterwards. The alloys annealed at 800 °C have relatively fine precipitation and microstructure, which could be responsible for their higher hardness. Afterwards, the fine precipitations become larger and the effect of the interface strengthening become smaller.

**Figure 6:**
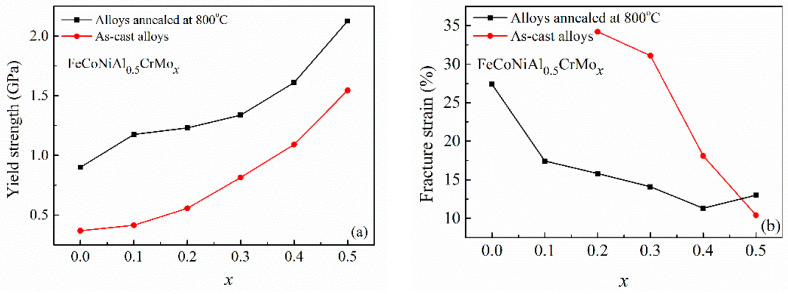
Yield strength (**a**) and fracture strain (**b**) of the as-cast alloys and the alloys annealed at 800 °C for 10 h.

Figure 6 presents the fracture strain and yield strength of the as-cast Al_0.5_CoCrFeMo_x_Ni high-entropy alloys and the alloys annealed at 800 °C for 10 h. It is clear that the fracture strain decreases, and the yield strength increases with Mo content increasing from 0 to 0.5. The alloys annealed at 800 °C have higher yield compressive strength and smaller compressive fracture strain than those of the as-cast alloys, which is attributed to the formation of fine (Al,Ni)-rich ordered BCC and (Cr,Mo)-rich σ phases. Both the Mo content and annealing processes have a great influence on the microstructure and mechanical properties of Al_0.5_CoCrFeMo_x_Ni high-entropy alloys. For the as-cast alloys, the Mo_0.3_ and Mo_0.4_ alloys can have balanced properties of compressive strength and ductility, while an appropriate annealing process can provide more chances to modify the mechanical properties of the alloys. For example, the Mo_0.5_ alloy annealed at 800 °C can have a high compressive yield strength up to 2.1 GPa (ultimate fracture strength of 2.6 GPa) with an accepted fracture strain of 13%. The Mo_0.1_ alloy annealed at 800 °C has a compressive yield strength of 1.2 GPa, an ultimate fracture strength of 2.2 GPa, and an ultimate fracture strain of 17%. Both the Mo content and annealing process can be used to tune the mechanical properties of the alloys.

## 4. Conclusions

The evolution of microstructure, phase and mechanical properties of the Al_0.5_CoCrFeMo_x_Ni high-entropy alloys have been investigated. Both the Mo content and annealing process can be used to tune the mechanical properties of the alloys. The following conclusions can be established from the current work.

(1) The annealing process at 600 °C for 10 h has no obvious effect on the microstructures of the Al_0.5_CoCrFeMo_x_Ni, while it can increase its hardness to some extent. The annealing at 800–1200 °C for 10 h causes the precipitation of (Al,Ni)-rich ordered BCC phase or/and (Cr,Mo)-rich σ phase, and greatly affects the microstructure and mechanical properties of the alloys.
(2) The evolution of structure with temperature can be classified into four types:
  (a) Mo_0_ alloy: mixed structure (FCC + BCC/B2).
  (b) Mo_0.1_ alloy: mixed structure (FCC + BCC/B2) below 600 °C → FCC + BCC/B2+ σ (800–1000 °C) → FCC + BCC/B2 (1100–1200 °C).
  (c) Mo_0.2_–Mo_0.3_ alloys: mixed structure (FCC + BCC/B2 + σ) below 1100 °C → FCC + BCC/B2 (1200 °C).
  (d) Mo_0.4_–Mo_0.5_ alloys: mixed structure (FCC + BCC/B2 + σ).
(3) The alloys annealed at 800 °C for 10 h have relatively finer microstructure and higher hardness and higher yield stress than the as-cast alloys, which can be attributed to the solid-state decomposition of FCC and B2 phases. The precipitations become larger at 1000–1100 °C.
(4) The morphology and amount of each phase in the alloys vary with the Mo content and annealing temperatures. The Mo_0.5_ alloy annealed at 800 °C have a high compressive yield strength up to 2.1 GPa with an accepted fracture strain of 13%.

## Figures and Tables

**Table 1:** Distribution of elements (at.%) in different regions of the alloys annealed at 1000 °C.

Alloys	Phases	Fe	Co	Ni	Al	Cr	Mo
Mo_0_-1000	DR: FCC	22.3	23.8	21.5	7.2	25.2	-
	ID: (Al,Ni)-rich BCC	11.0	15.5	35.5	30.6	7.4	-
	NLP: (Al,Ni)-rich BCC	16.0	18.6	22.4	31.2	11.8	-
Mo_0.1_-1000	DR: FCC	24.3	23.5	16.4	7.6	25.5	2.7
	ID: (Al,Ni)-rich BCC	14.8	19.8	22.6	30.9	10.8	1.1
	NLP: (Al,Ni)-rich BCC	16.8	16.8	28.5	20.7	15.7	1.6
	WP: (Cr,Mo)-rich σ	19.3	18.5	6.7	4.4	40.5	10.6
Mo_0.2_-1000	DR: FCC	23.7	21.0	20.6	8.2	21.2	5.3
	BP: (Al,Ni)-rich BCC	13.7	15.2	28.2	20.6	15.9	6.4
	WP: (Cr,Mo)-rich σ	20.9	20.5	14.9	6.0	28.1	9.6
	NLP: (Al,Ni)-rich BCC	20.2	22.9	21.9	11.0	19.6	4.5

**Table 2:** Distribution of elements (at.%) in different regions of the alloys annealed at 1000 °C.

Alloys	Regions	Fe	Co	Ni	Al	Cr	Mo
Mo_0.3_-1000	DR: FCC	22.8	18.8	21.3	10.1	20.7	6.3
	BP: (Al,Ni)-rich BCC	10.7	15.7	33.1	30.2	7.6	2.8
	WP: (Cr,Mo)-rich σ	18.1	18.6	7.7	3.6	30.3	21.6
Mo_0.4_-1000	DR: FCC	25.7	20.7	18.8	7.6	21.2	6.0
	BP: (Al,Ni)-rich BCC	9.3	17.5	30.6	25.0	11.5	6.1
	WP: (Cr,Mo)-rich σ	19.0	18.7	9.8	3.0	25.1	24.4
Mo_0.5_-1000	DR: FCC	23.3	21.3	21.1	7.0	17.7	9.5
	BP: (Al,Ni)-rich BCC	12.4	10.5	35.5	29.4	6.3	6.0
	WP1: (Cr,Mo)-rich σ	16.4	17.2	9.9	2.8	25.0	28.7
	WP2: (Cr,Mo)-rich	14.0	15.9	8.4	2.0	17.3	42.3

## References

[1] Cantor B., Chang I.T.H., Knight P., Vincent A.J.B. (2004). Microstructural development in equiatomic multicomponent alloys. Mater. Sci. Eng. A.

[2] Yeh J.W., Chen S.K., Lin S.J., Gan J.Y., Chin T.S., Shun T.T., Tsau C.H., Chang S.Y. (2004). Nanostructured high-entropy alloys with multiple principal elements: novel alloy design concepts and outcomes. Adv. Eng. Mater..

[3] Zhang Y., Zuo T.T., Tang Z., Gao M.C., Dahmen K.A., Liaw P.K., Lu Z.P. (2014). Microstructures and properties of high-entropy alloys. Prog. Mater. Sci..

[4] Tsai M.H. (2016). Three Strategies for the design of advanced high-entropy alloys. Entropy.

[5] Lu Z.P., Wang H., Chen M.W., Baker I., Jeh J.W., Liu C.T., Nieh T.G. (2015). An assessment on the future development of high-entropy alloys: Summary from a recent workshop. Intermetallics.

[6] Miracle D.B., Senkov O.N. (2017). A critical review of high entropy alloys and related concepts. Acta Mater..

[7] Wang W.R., Wang W.L., Wang S.C., Tsai Y.C., Lai C.H., Yeh J.W. (2012). Effects of Al addition on the microstructure and mechanical property of Al_x_CoCrFeNi high-entropy alloys. Intermetallics.

[8] He J.Y., Liu W.H., Wang H., Wu Y., Liu X.J., Nieh T.G., Lu Z.P. (2014). Effects of Al addition on structural evolution and tensile properties of the FeCoNiCrMn high-entropy alloy system. Acta Mater..

[9] Feng R., Gao M.C., Lee C., Mathes M., Zuo T., Chen S., Hawk J.A., Zhang Y., Liaw P.K. (2016). Design of Light-Weight High-Entropy Alloys. Entropy.

[10] Salishchev G.A., Tikhonovsky M.A., Shaysultanov D.G., Stepanov N.D., Kuznetsov A.V., Kolodiy I.V., Tortika A.S., Senkov O.N. (2014). Effect of Mn and V on structure and mechanical properties of high-entropy alloys based on CoCrFeNi system. J. Alloys Compd..

[11] Qiu Y., Hu Y.J., Taylor A., Styles M.J., Marceau R.K.W., Ceguerra A.V., Gibson M.G., Liu Z.K., Fraser H.L., Birbilis N. (2017). A lightweight single-phase AlTiVCr compositionally complex alloy. Acta Mater..

[12] Wang Z., Baker I., Guo W., Poplawsky J.D. (2017). The effect of carbon on the microstructures, mechanical properties, and deformation mechanisms of thermo-mechanically treated Fe_40.4_Ni_11.3_Mn_34.8_Al_7.5_Cr_6_ high entropy alloys. Acta Mater..

[13] Zhuang Y.X., Liu W.J., Chen Z.Y., Xue H.D., He J.C. (2012). Effect of elemental interaction on microstructure and mechanical properties of FeCoNiCuAl alloys. Mater. Sci. Eng. A.

[14] Zhou Y.J., Zhang Y., Wang Y.L., Chen G.L. (2007). Solid solution alloys of AlCoCrFeNiTi_x_ with excellent room-temperature mechanical properties. Appl. Phys. Lett..

[15] Tracy C.L., Park S., Rittman D., Zinkle S.J., Bei H., Lang M., Ewing R.C., Mao W.L. (2017). High pressure synthesis of a hexagonal close-packed phase of the high-entropy alloy CrMnFeCoNi. Nat. Commun..

[16] Ng C., Guo S., Luan J.H., Wang Q., Lu J., Shi S.Q., Liu C.T. (2014). Phase stability and tensile properties of Co-free Al_0.5_CrCuFeNi_2_ high entropy alloys. J. Alloys Compd..

[17] Wen L.H., Kou H.C., Li J.S., Chang H., Xue X.Y., Zhou L. (2009). Effect of aging temperature on microstructure and properties of AlCoCrCuFeNi high-entropy alloy. Intermetallics.

[18] Lin C.M., Tsai H.L., Bor H.Y. (2010). Effect of aging treatment on microstructure and properties of high-entropy Cu_0.5_CoCrFeNi alloy. Intermetallics.

[19] Jones N.G., Izzo R., Mignanelli P.M., Christofidou K.A., Tone H.J. (2016). Phase evolution in an Al0.5CrFeCoNiCu high entropy alloys. Intermetallics.

[20] Zhuang Y.X., Xue H.D., Chen Z.Y., Hu Z.Y., He J.C. (2013). Effect of annealing treatment on microstructures and mechanical properties of FeCoNiCuAl high entropy alloys. Mater. Sci. Eng. A.

[21] Wang W.R., Wang W.L., Yeh J.W. (2014). Phases, microstructure and mechanical properties of Al_x_CoCrFeNi high entropy alloys at elevated temperatures. J. Alloys Compd..

[22] Lu Y., Dong Y., Guo S., Liang L., Kang H., Wang T., Wen B., Wang Z., Jie J., Cao Z. (2014). A promising new class of high-temperature alloys: Eutectic high-entropy alloy. Sci. Rep..

[23] Gao X., Lu Y., Zhang B., Liang N., Wu G., Sha G., Liu J., Zhao Y. (2017). Microstructural origins of high strength and high ductility in an AlCoCrFeNi_2.1_ eutectic high-entropy alloy. Acta Mater..

[24] Li Z., Pradeep K.G., Deng Y., Raabe D., Tasan C.C. (2016). Metastable high-entropy dual-phase alloys overcome the strength-ductility trade-off. Nature.

[25] Li Z., Tasan C.C., Pradeep K.G., Raabe D. (2017). A TRIP-assisted dual-phase high-entropy alloy: Grain size and phase fraction effects on deformation behavior. Acta Mater..

[26] Zhuang Y.X., Zhang X.L., Gu X.Y. (2018). Effect of molybdenum on phases, microstructure and mechanical properties of Al_0.5_CoCrFeMo_x_Ni high entropy alloys. J. Alloys Compd..

[27] Gwalani B., Gorsse S., Choudhuri D., Styles M., Zheng Y., Mishra R.S., Banerjee R. (2018). Modifying transformation pathways in high entropy alloys or complex concentrated alloys via thermo-mechanical processing. Acta Mater..

[28] Lin C.M., Tsai H.L. (2011). Evolution of microstructure, hardness, and corrosion properties of high-entropy Al_0.5_CoCrFeNi alloy. Intermetallics.

[29] Rao J.C., Diao H.Y., Ocelik V., Vainchtein D., Zhang C., Kuo C., Tang Z., Guo W., Poplawsky J.D., Zhou Y. (2017). Secondary phases in Al_x_CoCrFeNi high-entropy alloys: An in-situ TEM heating study and thermodynamic appraisal. Acta Mater..

[30] Choudhuri D., Gwalani B., Gorsse S., Mikler C.V., Ramanujan R.V., Gibson M.A., Banerjee R. (2017). Change in the primary solidification phase from fcc to bcc-based B2 in high entropy or complex concentrated alloys. Scr. Mater..

[31] Tsai M.H., Chang K.C., Li J.H., Tsai R.C., Cheng A.H. (2016). A second criterion for sigma phase formation in high entropy alloys. Mater. Res. Lett..

